# Myeloid-driven immunosuppression as a context-dependent therapeutic bottleneck in pancreatic ductal adenocarcinoma

**DOI:** 10.3389/fimmu.2026.1936958

**Published:** 2026-10-07

**Authors:** Kai Sun, Lei Yang, Jingjing Li, Bo Jia, Min Fu, Qijia Zhang, Wei Yang

**Affiliations:** 1 Department of Hepatobiliary and Pancreatic Surgery, Beijing Tsinghua Changgung Hospital, School of Clinical Medicine, Tsinghua Medicine, Tsinghua University, Beijing, China; 2 School of Biomedical Engineering, Tsinghua University, Beijing, China; 3 Department of Liver Intensive Care Unit, Beijing Tsinghua Changgung Hospital, School of Clinical Medicine, Tsinghua Medicine, Tsinghua University, Beijing, China

**Keywords:** dendritic cells, immune checkpoint blockade, immunotherapy, myeloid-derived suppressor cells, pancreatic ductal adenocarcinoma, tumor-associated macrophages

## 1 Introduction

Pancreatic ductal adenocarcinoma (PDAC) remains one of the most treatment-refractory solid tumors despite modern multi-agent chemotherapy (1, 2). Immune checkpoint blockade has little effect outside the uncommon mismatch-repair-deficient or microsatellite-instability-high subgroup, and randomized studies have not established a reproducible survival advantage for immunotherapy-containing chemotherapy regimens in unselected disease (3, 4). Yet PDAC is not uniformly incapable of generating antitumor immunity. Microsatellite-stable tumors with high mutational burden and substantial immune infiltration exist; neoantigen quality is associated with long-term survival; and personalized RNA vaccination can induce durable, polyfunctional neoantigen-specific T cells (5–8). The recurrent problem is therefore not simply the absence of immunity but the failure to convert immune recognition into sustained tumor control.

We propose that suppressive myeloid programs should be treated as context-dependent therapeutic bottlenecks rather than universal targets. “Dominant” does not mean that a myeloid program is the only abnormality, nor can it be inferred from a single pretreatment association. It denotes a defect for which convergent evidence suggests a rate-limiting role at a specified step of the cancer-immunity cycle and for which selective correction or functional bypass is predicted to restore that step. Myeloid abundance may instead reflect tumor burden, tissue injury, infection, or chemotherapy. A credible myeloid-directed strategy therefore requires an explicit causal hypothesis, a matching intervention, and prespecified pharmacodynamic evidence while preserving essential host-defense functions.

## 2 Defining the dominant immune bottleneck

The PDAC myeloid compartment includes tumor-associated macrophages, monocytic and polymorphonuclear myeloid-derived suppressor cells (MDSCs), neutrophils, and dendritic-cell subsets. These populations are plastic and functionally heterogeneous (9–14). CD68 or CD163 staining can indicate macrophage abundance but cannot, by itself, establish suppressive function; similarly, a circulating MDSC count is not a surrogate for the intratumoral state unless prospectively validated. Reports should therefore distinguish three dimensions: abundance, spatial distribution, and functional state. Functional evidence may include antigen-presentation competence, suppressive metabolites or cytokines, ex vivo suppression, phagocytic activity, or validated transcriptional programs. The binary M1/M2 nomenclature should be used cautiously because it compresses continuous and context-dependent macrophage states into categories that are not reliably predictive of function (9–11). Spatial relationships among dendritic, myeloid, and T-cell populations can add information that cell density alone misses (15, 16).

Operationally, we consider a putative myeloid bottleneck “dominant” only when three conditions are largely met (**Table 1**). First, concordant evidence from at least two dimensions—preferably including function or spatial organization—should link the myeloid program to failure of a specified immune step, such as deficient cross-presentation, exclusion of tumor-reactive lymphocytes, or suppression of intratumoral effector activity. Preclinical PDAC data showing that antigen-presenting cDC1s can enable checkpoint responsiveness illustrate why priming competence should be tested functionally rather than inferred from cell density (17). Second, major competing explanations should be assessed, including inadequate antigenicity, absence of tumor-reactive clones, HLA loss or antigen-presentation defects in tumor cells, tumor-intrinsic interferon resistance, corticosteroid exposure, infection, biliary obstruction, treatment-related inflammation, and sampling bias. Third, when feasible, selective perturbation should produce proximal target engagement followed by recovery of the prespecified downstream immune process. Baseline profiling can therefore nominate a provisional bottleneck; serial perturbational evidence is what strengthens the claim that the process was rate-limiting rather than merely associated with resistance.

**Table 1:** Operational definition of dominant immune bottlenecks and hierarchy of selected clinical evidence in PDAC.

Panel A. Working bottleneck assignments		
Bottleneck and minimum evidence	Mechanism-matched strategy	Prespecified pharmacodynamic readout
Defective priming: low/immature cDC1 states or impaired cross-presentation, with weak tumor-reactive T-cell priming.	CD40 or innate-sensing agonism when biologically justified; dendritic-cell or neoantigen vaccination when an antigenic substrate is available.	cDC1 maturation and antigen-presentation programs; expansion/function of antigen-specific or experimentally tumor-reactive T cells.
Myeloid-associated trafficking/stromal exclusion: concordant evidence links a defined myeloid program to suppressive-cell localization at vascular or tumor-stromal interfaces, chemokine mismatch, abnormal perfusion, or increased tumor-cell-to-T-cell distance.	Selective chemokine-axis blockade, state reprogramming, or vascular/stromal remodeling while preserving dendritic cells and host defense.	Reduced tumor-cell-to-T-cell distance; tumor-reactive clones entering tumor nests; improved perfusion/adhesion; absence of compensatory suppressive influx.
Local effector suppression: tumor-reactive lymphocytes are present but impaired, with concordant ARG1, ROS, adenosine, IL-10, TGF-beta, inhibitory-ligand, or functional suppression evidence.	State-selective macrophage/MDSC modulation; adenosine/CD73, ARG1, or other pathway inhibition; checkpoint blockade only when a relevant lymphocyte substrate exists.	Proximal pathway inhibition plus restored tumor-reactive T-cell function; mechanism-specific NK, phagocytic, vascular, or stromal readouts when intended.
Systemic myeloid suppression: circulating monocytic/granulocytic expansion with demonstrated suppressive function and a concordant tissue/inflammatory signal; exclude infection, biliary obstruction, corticosteroids, and treatment effects.	Recruitment blockade or state modulation only when the axis is active; avoid broad depletion and pair with correction of an intratumoral barrier when present.	Functional suppression assay, concordant tissue change, preserved antimicrobial competence, and prospective pulmonary/infection surveillance.

Panel B. Selected clinical evidence		
Category, study, and evidence level	Key finding	Evidentiary interpretation
Direct myeloid targeting – PF-04136309 (18), phase Ib single-arm (n=21)	Peripheral CD14+CCR2+ monocytes decreased; incremental clinical benefit was not established in this uncontrolled study; pulmonary toxicity occurred in 24%.	Target engagement did not establish added efficacy and raised an important pulmonary safety signal.
Myeloid functional modulation – PRINCE (4), randomized phase II (n=105; historical arm benchmarks)	Only nivolumab/chemotherapy met its prespecified 1-year OS benchmark; immune correlates differed by arm.	No chemotherapy-only control and no definitive between-arm power; CD40 ineffectiveness cannot be concluded. Correlates are exploratory.
Myeloid functional modulation – entinostat (19), phase II single-arm (27 evaluable)	ORR 11%; primary endpoint not met; only four week-6 tumors informed late combination tissue pharmacodynamics.	Myeloid/transcriptional changes may coexist with limited efficacy; the small on-treatment tissue set constrains inference.
Myeloid functional modulation – RESOLVE (20), randomized double-blind phase III (n=424)	Ibrutinib plus chemotherapy did not improve OS; PFS and ORR were inferior, with lower treatment exposure.	High-level negative evidence against unselected BTK addition; the immunomodulatory rationale did not translate into clinical benefit.
Myeloid functional modulation – ARC-8 (21), phase Ib with randomized expansion between two quemliclustat regimens	Paired-biopsy NR4A downregulation was associated with T-cell activation and survival.	No concurrent chemotherapy-only arm isolated quemliclustat benefit; NR4A analyses were *post hoc*/exploratory.
Non-myeloid therapy with myeloid/spatial correlates – toripalimab (22), phase Ib/II single-arm (n=72)	Spatial model used 149 regions from 47 patients; mean AUC was approximately 0.80.	No chemotherapy control; region-level partitioning creates potential within-patient leakage unless patient grouping was enforced. Patient-level external validation is required.

Panel A presents working, testable assignments rather than validated clinical subtypes; multiple bottlenecks may coexist. Panel B separates direct myeloid targeting, myeloid functional modulation, and non-myeloid therapy with myeloid/spatial correlative analyses, while distinguishing randomized from single-arm evidence. AUC, area under the curve; BTK, Bruton tyrosine kinase; cDC1, type 1 conventional dendritic cell; MDSC, myeloid-derived suppressor cell; ORR, objective response rate; OS, overall survival; PFS, progression-free survival; ROS, reactive oxygen species.

Anatomical site should be incorporated into mechanistic assessment. Matched single-cell and spatial studies show that liver metastases can contain macrophage and neutrophil states distinct from primary pancreatic tumors, while human and experimental data support organ-specific microenvironmental remodeling, including a specialized immunomodulatory peritoneal niche (12, 23–27). A biomarker from a primary lesion should therefore not automatically be used to select therapy for a progressing liver or peritoneal metastasis. Pretreatment and on-treatment biopsies should be obtained from comparable sites whenever feasible, with the sampled lesion, interval local therapy, biliary instrumentation, and recent systemic treatment documented. A practical schedule is baseline, an early pharmacodynamic time point selected based on the drug’s biology, first radiological assessment, and progression. Matched blood sampling is useful for kinetics and safety but should not replace tissue unless prospectively validated as a surrogate.

Myeloid-directed interventions can also produce antitumor effects that are not mediated by restoration of T-cell function. Relevant alternatives include NK-cell activation, macrophage-mediated phagocytosis (28), vascular normalization, altered stromal architecture, and changes in antigen release or presentation. Accordingly, failure to recover tumor-reactive T-cell activity should not render an entire intervention “mechanistically negative”. It means that a prespecified T-cell-restoration mechanism is unsupported. If another mechanism is proposed, it requires its own prespecified readout rather than retrospective reinterpretation after the trial (**Figure 1**).

**Figure 1:**
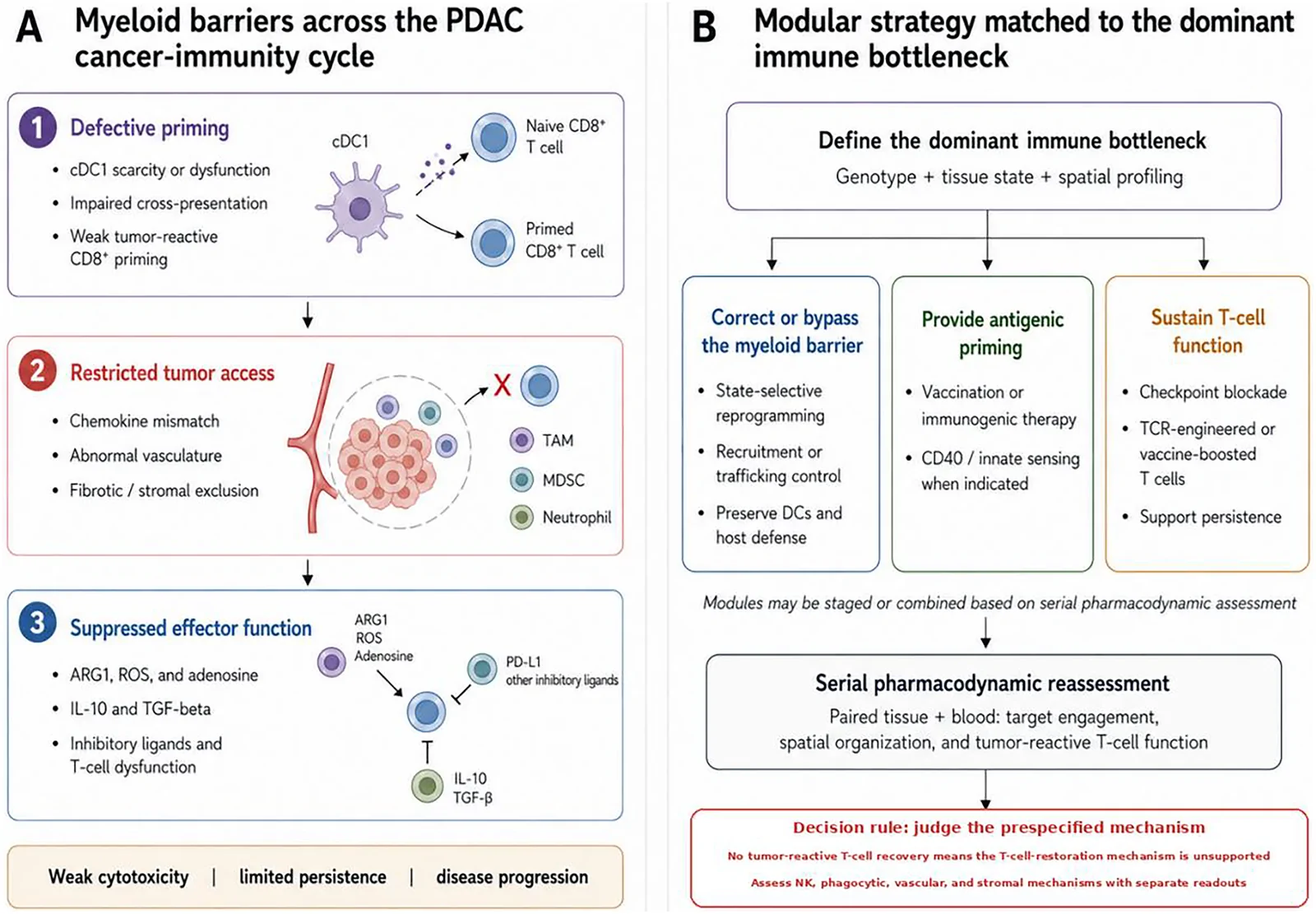
Myeloid barriers across the PDAC cancer-immunity cycle and a modular therapeutic framework. **(A)** Suppressive myeloid programs can impair cDC1-dependent priming, restrict lymphocyte access, and suppress intratumoral effector function. **(B)** Treatment should be matched to the dominant bottleneck. Correction or functional bypass of the myeloid barrier, antigenic priming, and lymphocyte support may be staged or combined according to serial pharmacodynamic assessment. Paired tissue and blood analyses should establish proximal target engagement and the downstream immune process specified *a priori*. If a therapy is intended to restore T-cell function but tumor-reactive T-cell activity does not recover, that prespecified mechanism is unsupported; this does not exclude antitumor effects through NK cells, macrophage phagocytosis, vascular remodeling, or stromal remodeling. ARG1, arginase 1; cDC1, type 1 conventional dendritic cell; DC, dendritic cell; MDSC, myeloid-derived suppressor cell; PDAC, pancreatic ductal adenocarcinoma; PD-L1, programmed death-ligand 1; ROS, reactive oxygen species; TAM, tumor-associated macrophage; TCR, T-cell receptor.

## 3 What the clinical evidence establishes

We performed a focused, non-systematic search of MEDLINE via PubMed, supplemented by backward reference screening, with a search cutoff of 31 July 2026. We prioritized peer-reviewed PDAC clinical studies that directly targeted myeloid recruitment or function, deliberately modulated antigen-presenting or myeloid states, or included informative tissue-based myeloid/spatial analyses. Evidence from randomized controlled studies was weighted above uncontrolled activity signals, and *post hoc* biomarker analyses were treated as hypothesis-generating unless independently validated. This was an evidence-focused Opinion rather than a systematic review, so **Table 1** is deliberately representative rather than exhaustive.

Available randomized data do not support empirical immunotherapy combinations in unselected patients. In PA.7, adding durvalumab and tremelimumab to gemcitabine plus nab-paclitaxel did not improve overall survival, but the trial did not identify which immune step remained rate-limiting (3). PRINCE is more nuanced than a simple positive-versus-negative comparison: the study had no chemotherapy-only control, each experimental arm was assessed against a historical 1-year overall survival benchmark, and the study was not powered to establish superiority between arms. Only the nivolumab/chemotherapy arm met its prespecified benchmark, whereas the sotigalimab-containing arms did not; this does not establish that CD40 agonism is ineffective (4). Arm-specific immune associations are biologically interesting but remain exploratory. Likewise, single-arm CD40 studies, including early sotigalimab studies, neoadjuvant selicrelumab, and OPTIMIZE-1, demonstrate tissue remodeling or encouraging activity without isolating the incremental clinical contribution of CD40 agonism (29–31).

These studies indicate that pharmacodynamic activity alone is insufficient to establish clinical benefit. Entinostat plus nivolumab altered dendritic-cell and inflammatory programs, yet the phase II primary endpoint was not met. Importantly, only four week-6 tumor samples informed the late combination pharmacodynamic analysis, limiting tissue-level inference (19). Toripalimab plus gemcitabine/nab-paclitaxel showed single-arm activity and an exploratory spatial model based on 149 regions from 47 patients (22). Because the published development split was region-based and patient-level grouping was not clearly specified, regions from the same patient may have entered different data partitions; within-patient leakage therefore cannot be excluded, and patient-level external validation is required before clinical use. In ARC-8, CD73 inhibition was associated with NR4A downregulation and T-cell activation in paired biopsies, but these were *post hoc* exploratory biomarker analyses and no concurrent chemotherapy-only arm isolated the incremental effect of quemliclustat (21).

Negative studies also place important constraints on this framework. The CCR2 inhibitor PF-04136309 reduced circulating CD14-positive CCR2-positive inflammatory monocytes, yet did not establish an incremental clinical benefit over gemcitabine/nab-paclitaxel in this uncontrolled phase Ib study and produced pulmonary toxicity in 24% of 21 patients, including severe pneumonitis or hypoxia and a fatal pneumonia (18). In the randomized, double-blind phase III RESOLVE trial, adding the BTK inhibitor ibrutinib to gemcitabine/nab-paclitaxel did not improve overall survival; progression-free survival and response rate were worse, and treatment exposure was lower (20). These examples show why a change in cell number or pathway activity cannot be equated with therapeutic causality. The targeted program may not have been dominant, the magnitude or tissue location of modulation may have been inadequate, compensatory suppressive circuits may have emerged, or toxicity may have reduced effective therapy. Myeloid-directed trials should therefore prospectively monitor pulmonary events, infection, cytopenias, and preservation of normal antimicrobial defense alongside tumor pharmacodynamics.

Together, the literature supports an evidence hierarchy rather than a binary claim that “myeloid targeting works” or “does not work”. Randomized evidence of benefit with mechanism-consistent pharmacodynamics provides the strongest support; clinical benefit without mechanistic confirmation supports efficacy but not the proposed pathway; pharmacodynamic change without benefit proves perturbation only; and *post hoc* associations remain biomarker hypotheses. **Table 1** applies this hierarchy while separating direct myeloid targeting, myeloid functional modulation, and non-myeloid therapies with myeloid or spatial correlates.

## 4 Principles for mechanism-matched trials

Trial entry should require evidence that the targeted axis is active in the lesion that will be used to judge mechanism. Defective dendritic-cell priming may justify CD40 or innate-sensing approaches or vaccination when an antigenic substrate is available; myeloid-associated spatial exclusion may justify selective trafficking or vascular or stromal intervention; and local effector suppression may justify pathway-specific inhibition when tumor-reactive lymphocytes are already present. These categories are working hypotheses, not validated PDAC subtypes, and more than one bottleneck may coexist. A complete regimen may therefore need antigenic priming, correction or bypass of the dominant suppressive barrier, and sustained lymphocyte support. RNA vaccination and adoptive T-cell therapies demonstrate that a tumor-reactive repertoire can be generated or introduced in PDAC, but they do not by themselves establish that a myeloid barrier has been corrected (7, 8, 32, 33).

Pharmacodynamic plans should explicitly separate proximal target engagement from downstream rescue. For a recruitment inhibitor, proximal evidence might be reduced trafficking of the intended suppressive population into the sampled lesion; for an adenosine-directed agent, it might be suppression of a prespecified pathway signature; for macrophage reprogramming, it should be a functionally interpretable state change rather than a reduction in CD163 alone. Downstream readouts should then test the biological consequence: improved cross-presentation, shorter tumor-cell-to-T-cell distance, expansion of experimentally tumor-reactive clones, cytokine production, or cytotoxicity. NK-cell, phagocytic, vascular, and stromal hypotheses require corresponding measures of NK activation and killing, tumor-cell engulfment, perfusion or endothelial adhesion, and predefined matrix/spatial remodeling. Such prespecification reduces the risk of *post hoc* mechanistic attribution based on whichever immune variable changes.

Sampling design should likewise be prespecified. Whenever clinically safe, the same lesion—or at minimum the same anatomical compartment—should be sampled at baseline and on treatment. The early biopsy should capture expected target engagement before radiographic selection obscures causality; the first imaging assessment should determine whether the biological change is translating into tumor control; and the progression biopsy can reveal pathway escape or a new bottleneck. Parallel blood measurements can identify systemic pharmacodynamics and toxicity, but blood–tissue discordance should be retained as biological information. In multi-component regimens, a clinical response should not be assigned to the myeloid agent without an appropriate control or a compelling, prospectively defined mechanistic chain.

Biomarker-enriched randomized designs are ultimately needed. Enrichment should be based on a composite that captures the proposed biology—tumor genotype where relevant, myeloid functional state, spatial organization, dendritic-cell competence, and the presence and fitness of tumor-reactive lymphocytes—rather than a single density marker. Safety should also inform mechanism-matched intervention selection because myeloid cells contribute to antimicrobial defense, wound repair, and tissue homeostasis (9–11). Accordingly, informative trials should prioritize identifying a plausible bottleneck, modifying it selectively and safely, demonstrating the prespecified downstream mechanism, and then testing whether that mechanistic rescue improves clinical outcome.

## 5 Discussion

Myeloid suppression in PDAC is neither universal nor adequately described by a single marker. “Dominant immune bottleneck” should be a testable, patient- and lesion-specific causal hypothesis rather than a fixed taxonomy. The framework requires distinguishing association from rate limitation, controlling for anatomical site and biopsy timing, and separating target engagement from functional rescue. If tumor-reactive T-cell recovery was the prespecified mechanism and does not occur, that mechanism is unsupported; NK, phagocytic, vascular, or stromal activity must be judged by their own endpoints. The goal is selective correction or bypass of the process actually limiting antitumor immunity, followed by randomized validation that mechanistic rescue improves patient outcomes.
